# Removal of a Pencil Embedded in a Child’s Foot: A Case Report

**DOI:** 10.7759/cureus.20033

**Published:** 2021-11-30

**Authors:** Koshi Ota, Hiroki Yokoyama, Akira Takasu

**Affiliations:** 1 Emergency Medicine, Osaka Medical and Pharmaceutical University, Takatsuki, JPN

**Keywords:** foreign body, foot, children, autism spectrum disorder, attention-deficit hyperactivity disorder

## Abstract

The foot is the most common anatomic site for foreign body embedment in both children and adults. An 11-year-old boy boy with a history of autism spectrum disorder (ASD), learning disorder, and attention-deficit hyperactivity disorder (ADHD) was brought to our ED with a pencil deeply embedded in his right foot. The broken portion of the pencil was completely embedded in his right foot, with mild bleeding and it could not be extracted easily. The pencil was eventually mobilized via gentle back-and-forth twisting motion, which allowed successful removal of a significant portion of the embedded pencil.

To establish the presence of a foreign body, as in each X-ray, the affected body part should be imaged in at least two directions. Based on the density of the embedded foreign body, ultrasound imaging should be considered.

## Introduction

By virtue of its anatomic location, the foot is susceptible to penetrating injury from the ground when bare, hence, the foot is the most common anatomic site for foreign body embedment in both children and adults [1,2]. For physicians treating patients with pencil as the impaling foreign body, it is incumbent for the examiner not to overlook pencil remnant because it is made of brittle wood that can break off easily. Additionally, the intactness of the pencil tip should be confirmed after removal. Here, we describe an 11-year-old autistic boy with a pencil embedded in his foot. The patient and his mother have given their permission to publish the features of his case, and the identity of the patient has been shielded for patient's confidentiality.

## Case presentation

**Figure 1:**
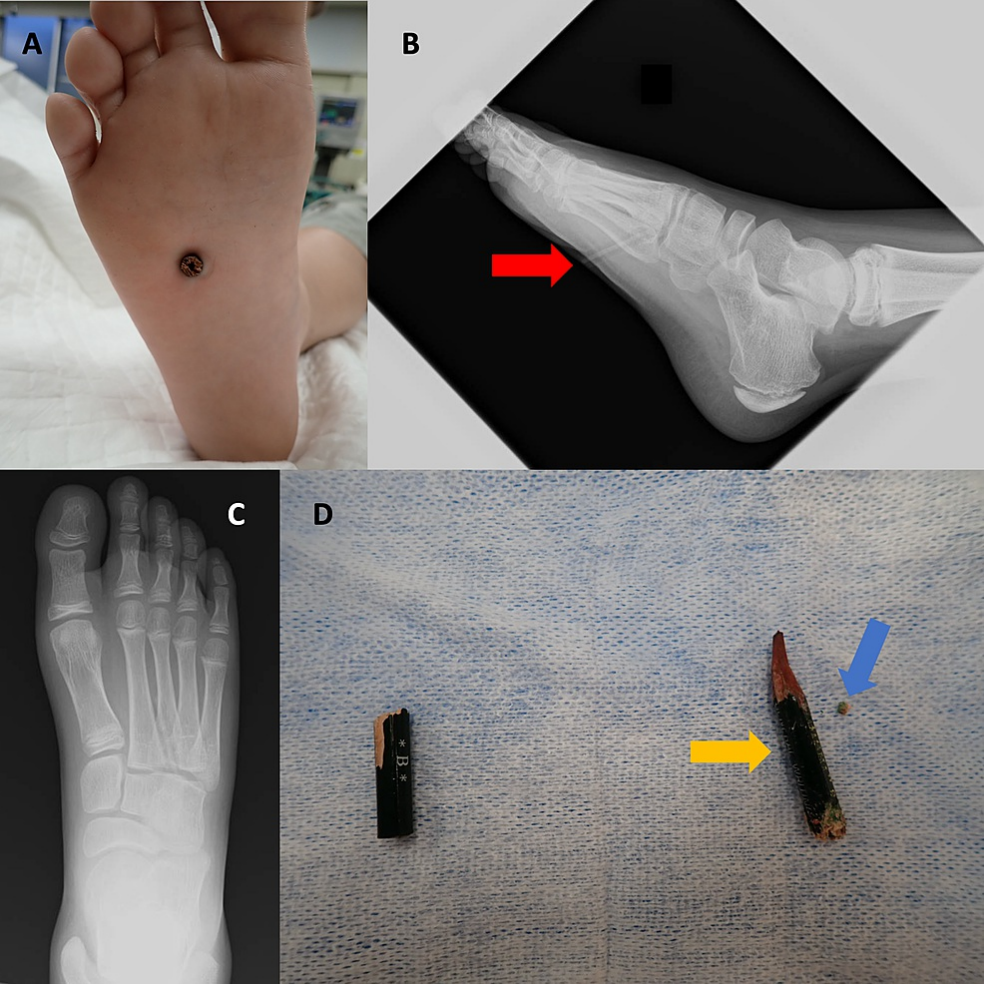
A pencil embedded in a child’s foot. 1A) The end of the pencil was visible on the sole of the right foot; 1B) Lateral X-ray of the right foot. A stick-like foreign body is seen in the right foot (red arrow); 1C) Anteroposterior X-ray of the right foot. The foreign body was not visible; 1D) The fragment on the left is the broken external part of the pencil and the fragment on the right is the part that was embedded in the foot (orange arrow). The smaller right-most fragment is the part that broke off when trying to remove the pencil using mosquito forceps (blue arrow).

An 11-year-old boy, weighing 50 kg, poorly commutative with a history of autism spectrum disorder (ASD), learning disorder, and attention-deficit hyperactivity disorder (ADHD), was brought to our ED with a pencil embedded in his right foot. His full-scale intelligence quotient (FSIQ) was 76, and his intellectual function is further impaired by an inability to concentrate at times. He, apparently, was feeling testy and irritated at school, so he began to stab at his shoe repeatedly with a pencil, resulting in the pencil piercing through the sole of the shoe and impaling his foot. His vital signs on arrival at the ED were normal. The broken portion of the pencil was completely embedded in his right foot, with only mild bleeding and soft tissue trauma (Figure 1A). The pencil could not be extracted readily because it was completely embedded within the foot, with the outer edge nearly flush with the skin, and he complained of significant pain. Plain film radiograph (X-ray) of the right foot revealed a stick-like foreign body in the right foot (Figure 1B), although the anterior (AP) view did not show the foreign body (Figure 1C). The patient was anesthetized with 30 mg ketamine hydrochloride administered intravenously to allow for removal of the pencil. However, the pencil could not be extracted easily because it was embedded and wedged deeply, and only a small fragment could be removed with mosquito forceps (Figure 1D). The pencil was then twisted in a gentle back-and-forth motion, which successfully freed up the large piece of the broken pencil (Figure 1D). After confirming that the tip of the removed pencil was intact, thorough lavage of the site was performed. Ultrasound of the right foot did not show evidence of any pencil remnant. The boy was able to walk without pain or disability at his follow-up in clinic one week later.

## Discussion

The foot is the most common anatomic site for foreign body embedment in both children and adults [1,2]. As a caveat for physicians treating patients with pencil as the impaling and embedded foreign body, it is easy to overlook a pencil remnant because it is usually made of brittle wood and is susceptible to break off with minimal force. Additionally, the intactness of the tip of the pencil should be confirmed after removal. Although an ultrasound-guided hydro-dissection technique has recently been introduced for the removal of foreign bodies [3], this technique was not indicated in this case as the end of the pencil was clearly visible on the sole of the foot. One study demonstrated that foreign bodies could be seen by using oblique and lateral views radiographs of the pelvis [4]. Our previous study also needed two directional views in order to reveal the exact location of small embedded acupuncture needles in an arm [5]. Table 1 shows the capability of various imaging modalities including X-ray, CT, MRI and USG in visualizing metal, wood, plastic, stone, and glass [6,7]. Different materials have unique physical properties such as density and compressiveness that maybe visualized more optimally by different imaging modality.

**Table 1:** The capability of X-ray, CT, MRI and USG in visualizing metal, wood, plastic, stone, and glass. +++: Excellent resolution of details and visibility, good demarcation from surroundings. ++: Good resolution of details and clear visibility, demarcation from surroundings. +: Insufficient resolution of details and visibility, insufficient demarcation. ±: Details not resolved and bad visibility, bad demarcation from surroundings. -: Invisible. *: Depend on the size of materials. All materials could be detected by using USG when fragments were more than 3 mm in size.

	Imaging modalities			
Materials	X-ray	CT	MRI	USG
Metal	+++	+++	+	*
Wood	-	-	-	*
Plastic	±	++	+	*
Stone	++	+++	++	*
Glass	+	+++	+	*

## Conclusions

To establish the presence of a foreign body, as in each X-ray film, the affected body part should be imaged in at least two directions. Depending on the type of embedded foreign body involved, USG should be utilized to help rule out embedded remnants.
